# Impact of old age, comorbidity, and polypharmacy on adjuvant endocrine therapy for patients with early-stage breast cancer

**DOI:** 10.1016/j.breast.2026.104949

**Published:** 2026-10-27

**Authors:** Chunhuan Lao, Marion Kuper-Hommel, Ian Campbell, Ineke Meredith, Abbey Wrigley, Ross Lawrenson

**Affiliations:** a School of Health Equity and Innovation, The University of Waikato, Hamilton, New Zealand; b Medical Oncology, Te Whatu Ora Waikato, Hamilton, New Zealand; c School of Medicine, The University of Auckland, Auckland, New Zealand; d Paris Breast Centre, Paris, France; e Canopy Cancer Care, Auckland, New Zealand; f School of Medicine, The University of Waikato, Hamilton, New Zealand

**Keywords:** Breast cancer, Adjuvant endocrine therapy, Polypharmacy, Comorbidities, Older patients

## Abstract

**Objectives:**

To explore effects of adjuvant endocrine therapy (ET) among women with early-stage hormone receptor positive breast cancer aged 70+ years, with comorbidities or experiencing polypharmacy.

**Methods:**

Women diagnosed with breast cancer in 2006-2022 were identified from the Te Rēhita Mate Ūtaetae – Breast Cancer Foundation National Register in New Zealand. Patients with pathological stage I-III, hormone receptor positive disease, and had surgery within 1 year after diagnosis were included. All-cause survival, breast cancer-specific survival and disease-free survival were explored between women with adjuvant ET and women without using Kaplan-Meier methods, after propensity score matching on key factors.

**Results:**

Only half patients completed 5 years of adjuvant ET, and the rate declined with cancer stage. Women with stage I cancer aged 70+ years, with comorbidities or experiencing polypharmacy had excellent relapse-free survival and breast-cancer specific survival but poor all-cause survival. The 10-year all-cause mortality rate (26.1%-37.2%) were more than 10 times the breast cancer-specific mortality rate (0.7%-3.2%) and more than three times the cancer relapse rate (6.0%-10.3%). Among these women, no significant difference was found in all-cause survival, breast cancer-specific survival or disease-free survival between those receiving and not receiving adjuvant ET.

**Conclusions:**

Women with stage I hormone receptor-positive breast cancer who were older, had comorbidities or polypharmacy showed excellent breast cancer-specific and disease-free survival but poorer all-cause survival. These patients with stage I cancer may derive limited benefit from adjuvant ET with high competing mortality. Further research should be performed to verify these findings.

## Highlights

• Only half patients completed 5 years of adjuvant ET with 1/5 stopped within 1 year.
• Five-year ET completion rate declined with cancer stage.
• Stage I breast cancer with old age/comorbidity/polypharmacy had low risk of relapse.
• Adjuvant endocrine therapy may offer limited benefit for these patients.
• All-cause mortality for them was 10 times the breast cancer-specific mortality.

## Graphical abstract

**Figure:**
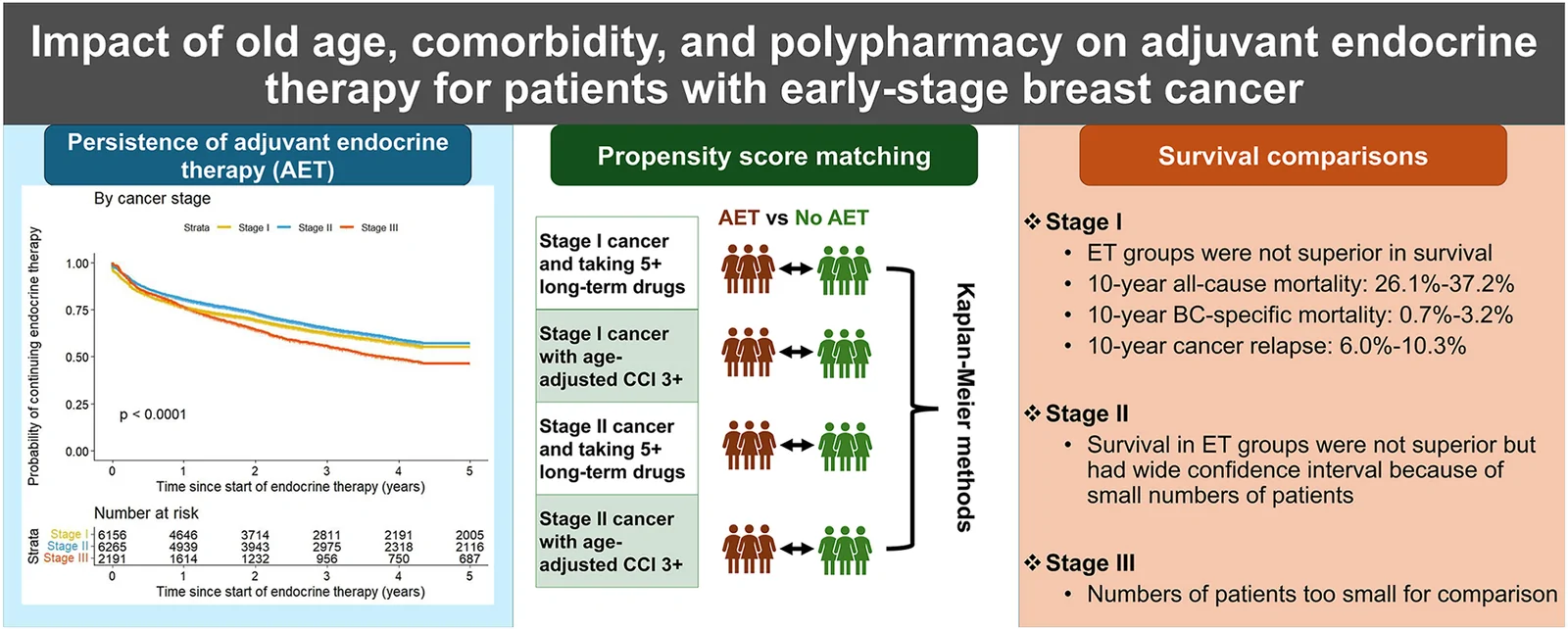

## 1 Introduction

Most patients with early-stage breast cancer are initially treated with surgery [1], and adjuvant treatments such as endocrine therapy (ET) which are used to reduce the risk of recurrence and to improve survival. Early-stage breast cancer is generally defined as stage I–III disease, with the overall stage determined using the TNM classification, which assesses primary tumour size and extent (T), regional lymph node involvement (N), and distant metastasis [2], [3]. Invasive tumours which are oestrogen receptor (ER) and/or progesterone receptor (PR) positive (+) are sensitive to ET, and patients with ER/PR + cancers can benefit from ET [4], [5], [6]. ET is usually prescribed for 5-10 years depending on the risk of cancer recurrence [7]. Due to the long duration of treatment and side effects, adherence to ET is a known challenge [8].

Many patients diagnosed with breast cancer are older and are likely to have comorbidities resulting in polypharmacy commonly defined as the use of five or more medicines at the same time [9], [10]. These patients can have a high incidence of experiencing drug-drug interactions, which may cause toxicity or reduced effectiveness of treatment. The impact of age, comorbidities and polypharmacy, on adjuvant ET for breast cancer remains under-researched globally. Yet these issues raise a lot of questions for clinicians and patients. Is adjuvant ET beneficial for older patients with multiple comorbidities who are exposed to polypharmacy?

An answer to these questions may be found by conducting a clinical trial among these patients. However, the effect of adjuvant ET on breast cancer outcomes takes many years to demonstrate. In addition, the difficulties in recruiting older patients, individuals with multiple comorbidities and/or those experiencing polypharmacy and randomising them into balanced groups, make it challenging to conduct clinical trials for these patients. In contrast, well-designed observational studies can offer valuable insights into long-term treatment effects in real-world settings. Large randomised trials in elderly patients with breast cancer are scarce, and there is little level I evidence for the treatment of such patients [11]. These patients are often underrepresented in clinical trials due to stringent eligibility criteria. This study aims to explore effects of adjuvant ET among elderly patients, with severe comorbidities or experiencing polypharmacy using real-world, population-based data from New Zealand (NZ).

## 2 Methods

### 2.1 Data sources

The study population was identified from the Te Rēhita Mate Ūtaetae Breast Cancer Register (BCFNR). The register is a national-level database of patients diagnosed with breast cancer in NZ. Participants are followed up each year to monitor how they are doing following their breast cancer diagnosis. It is a comprehensive resource for research and audit in NZ. It contains detailed information on patient demographics (e.g. date of birth, gender and comorbidities), cancer diagnosis (including date of diagnosis, cancer stage, cancer grade, tumour size and biomarker status), treatment (surgery, radiotherapy, ET, chemotherapy and biological therapy) and outcomes (local recurrence, distant metastasis, second primary cancer and death). For ET, the register records the referral for ET, reasons for not being referred (e.g. patient refused, patient unfit), the timing of ET (neo-adjuvant, adjuvant, local-recurrence and metastasis), start and end date and reason for stopping ET.

The register data were linked to the Pharmaceutical Collection (PHARMS) to investigate what medication patients were on before their breast cancer diagnosis, to the National Minimum Dataset to identify the burden of comorbidity, and to the Mortality Collection and Death Certificates for more updated data on mortality. The PHARMS dataset contains subsidised dispensing records including chemical name, therapeutic group, date and quantity dispensed. The National Minimum Dataset is a national collection of public and private hospital discharge information, including coded clinical data for same day and multi day inpatients. The Mortality Collection and Death Certificates record date and cause of death.

### 2.2 Study population

Women diagnosed with breast cancer between 01 July 2006 and 30 June 2022 were identified, and only those who had primary surgery within 1 year after diagnosis and had pathological stage I-III disease (TNM staging system, 8th edition) [3] were included. For exploring the effects of adjuvant ET on breast cancer survival, more exclusion criteria were applied: 1) patients developing metastatic disease within 4 months post-surgery (to minimise the risk of having metastatic disease at surgery); 2) patients who were considered not fit for ET and were not referred; 3) patients who started adjuvant ET more than one year after primary surgery; and 4) patients with missing data on key variables for propensity score matching. Patients who had only one dispensing record of ET (either neoadjuvant or adjuvant) or with the duration of ET less than 1 month were classified into the control group. This was to maximise the number of patients in the control group. Patients having neoadjuvant ET for 1 month or more and not having adjuvant ET were not included in the control nor the treatment group.

### 2.3 Age-adjusted charlson comorbidity index (CCI) score

The CCI score assigns weighted scores to chronic conditions based on their association with mortality risk [12]. The age-adjusted CCI score adds age points for patients aged 50 years or older: adding one point for age of 50-59 years; two points for age of 60-69 years; three points for age of 70-79 years and four points for age of 80 years or older [13]. For example, the age-adjusted CCI score of a 70-year-old woman without any significant comorbidities is three, therefore all women aged 70 and above have an age-adjusted CCI score 3+.

### 2.4 Definition of polypharmacy

Polypharmacy prior to cancer diagnosis was defined as the dispensing of five or more long-term medications (excluding those in Appendix Table 1) [9], [10]. The methods for identifying patients experiencing polypharmacy before cancer diagnosis were mostly based on the report on polypharmacy by the Health Quality & Safety Commission New Zealand [9]. As part of this, the number of long-term medications in the 6 months period leading up to breast cancer diagnosis was explored. Where medication dispensing records noted a patient was dispensed a ‘medication’ or ‘chemical’ for more than 90 days, this was considered a long-term medication. For medication dispensing records which did not disclose the number of days dispensed, but it had in fact been dispensed in the quarter before as well as the present quarter, then this was considered a long-term medication.

### 2.5 Probability of completing 5 years ET

The probability of finishing 5 years of adjuvant ET was explored with Kaplan-Meier curves displaying the time from start to end of ET by cancer stage, polypharmacy and age-adjusted CCI score. The differences were examined with Log-rank test. All patients were censored at 5 years after the commencement of ET or at the stop date if the patient did not finish the 5 years of ET. Reason for stopping adjuvant ET was analysed among patients who discontinued ET. For patients missing a reason for stopping adjuvant ET in the register, we compared the last date of ET with the date of cancer relapse (local, regional or distant recurrence, date of second primary breast cancer and date of death. If the last date of ET matched any of these dates, the patient was considered stopping adjuvant ET for cancer relapse, second primary cancer or death. The reasons for discontinuing adjuvant ET were stratified into three groups: cancer relapse (including cancer relapse and breast cancer death), other deaths, and other reasons (including adverse drug events, patient stopped without consulting clinician, other reasons, and unknown excluding cancer relapse and death).

### 2.6 Propensity score matching

Propensity score matching was performed to create comparable treatment and control groups, minimizing baseline differences between them. Propensity scores were estimated using a logistic regression model that included number of long-term drugs, age at diagnosis, CCI score (not age-adjusted), menopausal status (pre, peri or post-menopausal), ethnicity (Asian, Māori, Pacific, or European/Others), year of diagnosis, cancer grade, tumour size, human epidermal growth factor receptor 2 (HER2) status, type of surgery (breast conserving surgery or mastectomy), radiotherapy, chemotherapy and biological therapy as covariates. A 1:1 nearest-neighbour matching algorithm without replacement was applied, using a calliper of 0.02 to ensure a close match between the two groups. These analyses were only performed in patients with stage I-II breast cancer, because the number of patients with stage III breast cancer and not having adjuvant ET was too small. The effect of adjuvant ET on outcomes among patients with polypharmacy and patients with an age-adjusted CCI score of 3+ was explored after stratification by cancer stage (stage I and stage II). The propensity score matching was performed after these stratifications. To evaluate covariate balance, key variables were compared between the two groups before and after propensity score matching by calculating p-values and Standardized Mean Differences (SMD).

### 2.7 Survival analysis

After a matched treatment group and a matched control group were identified, the characteristics of the ET treatment and the no ET treatment group were compared before and after propensity score matching. ET exposure was treated as a fixed baseline classification rather than modelled as a time-dependent variable. The all-cause survival, breast cancer-specific survival and disease-free survival were examined between the two groups with Kaplan-Meier curves and Log-rank test. Survival probability and the 95% confidence interval (CI) at 2 years, 5 years and 10 years post-surgery were estimated. The survival analysis was from date of diagnosis to the date of event or the censor date which was 31 December 2023. The date of event was any death for all-cause survival, breast cancer death for breast cancer-specific survival, and local/regional recurrence, metastatic relapse, second primary breast cancer and breast cancer death for disease-free survival whichever is earlier.

All data analyses were conducted using R 4.0 (R Institute, Vienna, Austria). Approval for the study's ethics was obtained from the University of Waikato Human Research Ethics Committee, with the reference number HREC(Health)2024#45.

## 3 Results

During the study period, we identified 19,380 women who met the inclusion criteria (Fig. 1). After surgery, 14,612 (75.4%) women started adjuvant ET (including those with only one dispensing records). As shown in the Kaplan-Meier curves (Fig. 1), among women who started adjuvant ET after surgery, 54.7% (95% CI: 53.8%-55.6%) of patients finished the 5 years of adjuvant ET, with 21.8% (95% CI: 21.2%-22.5%) of women stopping adjuvant ET within 1 year of commencement. The probability of completing the 5 years treatment decreased with cancer stage (p-value<0.0001), with only 46.4% (95% CI: 44.2%-48.7%) of women with stage III cancer finished 5 years of adjuvant ET, compared to 55.2% (95% CI: 53.8%-56.5%) of women with stage I and 57.2% (95% CI: 55.9%-58.6%) of women with stage II cancer (Fig. 2). Patients with polypharmacy and patients with an age-adjusted CCI score 3+ were slightly less likely to complete the 5 years ET, but the difference was very small: 52.2% for patients with polypharmacy compared to 55.0% for patients without (p-value = 0.120) and 52.4% for those with an age-adjusted CCI score 3+ compared to 55.5% for others (p-value = 0.0068).

**Fig. 1:**
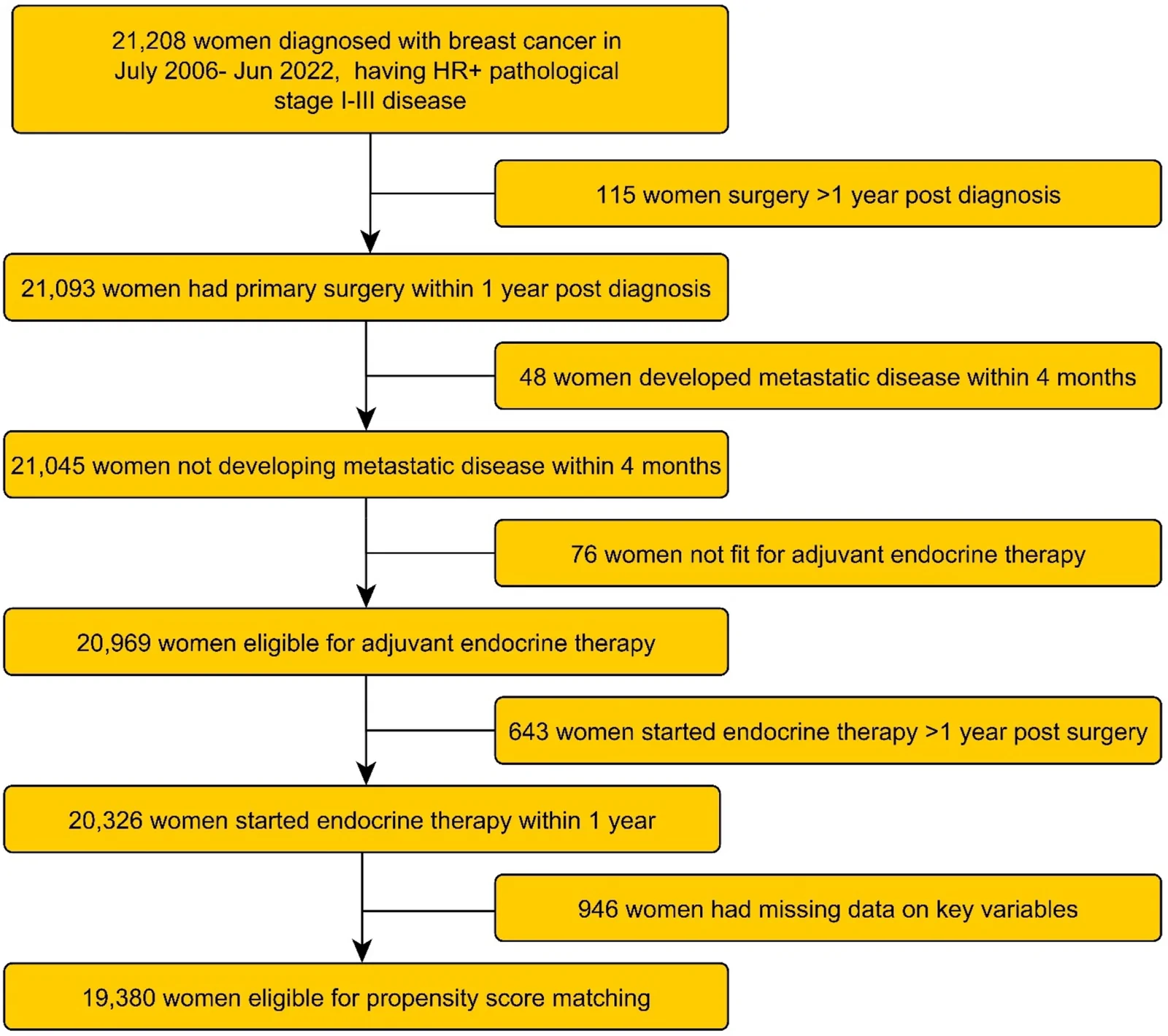
Flow diagram of patient inclusion in the study cohort.

**Fig. 2:**
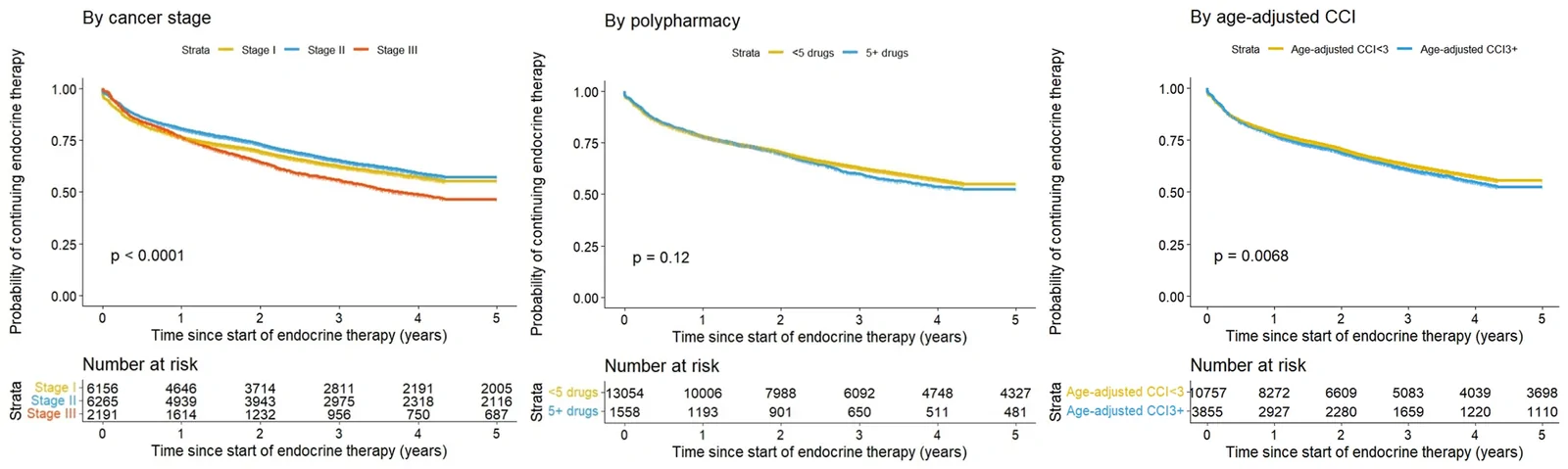
Time to end of endocrine therapy by cancer stage, polypharmacy and age-adjusted CCI score.

Among the 5925 women who discontinued adjuvant ET before the completion of 5 years, 495 (8.4%) patients stopped at cancer relapse (including second primary cancer and breast cancer deaths), 204 (3.4%) stopped at death from other causes, 5226 (88.2%) were due to other reasons (Table 1). Approximately one quarter of women with stage III cancer stopped adjuvant ET due to cancer relapse, compared to only 2.6% for women with stage I cancer and 8.2% of women with stage II cancer. Compared to their counterparts, patients with polypharmacy and patients with an age-adjusted CCI score of 3+ were more likely to have “Other deaths” as the reason of stopping ET and were less likely to be due to other reasons.

**Table 1:** Reason of stopping adjuvant endocrine therapy before completing the 5 years treatment.

Subgroup	Relapse^a^ n (%)	Other deaths n (%)	Other reasons^b^ n (%)	Total
**Duration**				
Discontinued within 1 month	8 (1.2)	3 (0.5)	649 (98.3)	660
Discontinued in 1∼12 months	137 (5.4)	45 (1.8)	2339 (92.8)	2521
Discontinued in 1∼3 years	256 (13.2)	103 (5.3)	1575 (81.4)	1934
Discontinued in 3∼5 years	94 (11.6)	53 (6.5)	663 (81.9)	810
**Cancer stage**				
Stage I	65 (2.6)	64 (2.6)	2362 (94.8)	2491
Stage II	194 (8.2)	86 (3.6)	2089 (88.2)	2369
Stage III	236 (22.2)	54 (5.1)	775 (72.8)	1065
**Polypharmacy**				
<5 long-term drugs	431 (8.2)	115 (2.2)	4725 (89.6)	5271
5+ long-term drugs	64 (9.8)	89 (13.6)	501 (76.6)	654
**Age-adjusted CCI score**				
<3	328 (7.6)	34 (0.8)	3947 (91.6)	4309
3+	167 (10.3)	170 (10.5)	1279 (79.1)	1616
**Total**	495 (8.4)	204 (3.4)	5226 (88.2)	5925

a Including local, regional and distant relapse, second primary cancer and breast cancer death.

b Including adverse drug events, patient's decision, other reasons and unrecorded reasons.

Before propensity score matching, there were significant difference between women receiving adjuvant ET and women not receiving adjuvant ET on key variables, including tumour size, HER2 status, cancer grade, and treatments, especially for stage I cancer (Appendix Table 2, 3, 4 and 5). After propensity score matching, we identified 299 women with stage I cancer with polypharmacy each in the control group and in the treatment group (Appendix Table 2), 743 women with stage I cancer and an age-adjusted CCI score of 3+ in each group (Appendix Table 3). The median time from surgery to initiation of adjuvant ET in the treatment group was 28 days for patients with polypharmacy and 30 days for patients with an age-adjusted CCI score of 3+. For stage II cancer, the respective number of women was 152 and 414 in each group (Appendix Tables 4 and 5). After propensity score matching, the distribution of patient characteristics and other treatments between the group receiving adjuvant ET and the group not receiving adjuvant ET was well balanced.

Women with stage I cancer with polypharmacy or with an age-adjusted CCI score of 3+ had excellent relapse-free survival and breast-cancer specific survival but very poor all-cause survival (Fig. 3, Table 2). The 10-year all-cause mortality rate (26.1%-37.2%) were more than 10 times the breast cancer-specific mortality rate (0.7%-3.2%) and more than three times the relapse rate (6.0%-10.3%) for these patients. Among women with stage I cancer with polypharmacy or with an age-adjusted CCI score of 3+, no significant difference was found in all-cause survival, breast cancer-specific survival or disease-free survival between patients receiving adjuvant ET and patients without. Among women with stage I cancer with polypharmacy, the 10-year breast cancer-specific survival and 10-year relapse free survival for those receiving adjuvant ET were 99.3% and 92.7% compared to 97.8% and 94.0% for patients without receiving adjuvant ET (p-value = 0.242 and 0.842, respectively). Among women with stage I cancer with an age-adjusted CCI score of 3+, the corresponding rates were 96.8% and 89.7% for those who received ET, versus 97.2% and 93.0% for those who did not (p-value = 0.839 and 0.701, respectively).

**Fig. 3:**
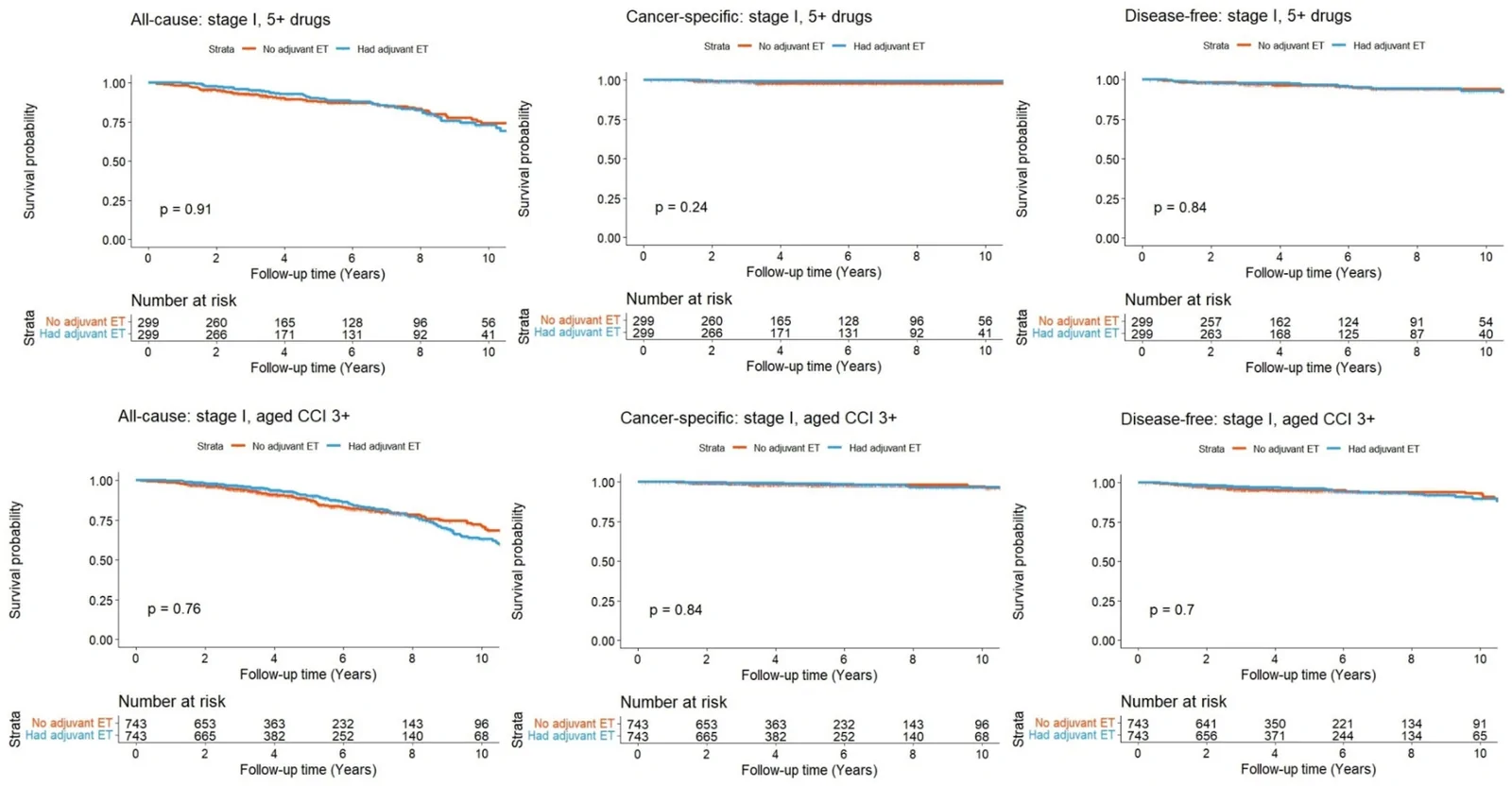
Survival in women with stage I cancer experiencing polypharmacy and women with an age adjusted CCI score 3+, comparing treatment and control groups.

**Table 2:** 2-, 5- and 10-year survival among women experiencing polypharmacy and women with an age adjusted CCI score 3+.

	5+ drugs			Age adjusted CCI score 3+		
	No adjuvant ET	Had adjuvant ET	p-value (Log-rank test)	No adjuvant ET	Had adjuvant ET	p-value (Log-rank test)
**Stage I**						
**All cause survival**						
2-year	95·6% (93·4%-98·0%)	97·6% (95·8%-99·4%)	0·912	96·7% (95·5%-98·0%)	98·1% (97·1%-99·1%)	0·759
5-year	87·7% (83·5%-92·1%)	90·3% (86·5%-94·4%)		87·5% (84·6%-90·5%)	90·2% (87·6%-93·0%)	
10-year	73·9% (66·8%-81·8%)	72·9% (65·5%-81·2%)		71·4% (66·0%-77·3%)	62·8% (56·3%-70·1%)	
**Breast cancer-specific survival**						
2-year	99·3% (98·4%-100·0%)	99·6% (99·0%-100·0%)	0·242	99·3% (98·7%-99·9%)	99·7% (99·3%-100·0%)	0·839
5-year	97·8% (96·0%-99·8%)	99·3% (98·3%-100·0%)		98·1% (96·9%-99·3%)	98·9% (97·9%-99·8%)	
10-year	97·8% (96·0%-99·8%)	99·3% (98·3%-100·0%)		97·2% (95·0%-99·4%)	96·8% (94·5%-99·1%)	
**Relapse-free survival**						
2-year	98·3% (96·8%-99·8%)	98·3% (96·9%-99·8%)	0·842	97·5% (96·4%-98·6%)	98·4% (97·4%-99·3%)	0·701
5-year	96·4% (94·0%-98·8%)	96·7% (94·4%-99·1%)		94·9% (93·1%-96·7%)	96·3% (94·7%-97·9%)	
10-year	94·0% (90·5%-97·6%)	92·7% (88·1%-97·5%)		93·0% (90·2%-96·0%)	89·7% (85·3%-94·4%)	

**Stage II**						
**All cause survival**						
2-year	92·1% (87·9%-96·5%)	96·7% (93·9%-99·6%)	0·166	92·9% (90·4%-95·4%)	97·3% (95·7%-98·9%)	0·022
5-year	71·9% (63·7%-81·1%)	80·4% (73·2%-88·4%)		75·7% (70·7%-81·1%)	83·1% (78·6%-87·9%)	
10-year	43·3% (30·9%-60·5%)	50·3% (38·6%-65·6%)		46·8% (38·2%-57·2%)	51·0% (42·1%-61·8%)	
**Breast cancer-specific survival**						
2-year	95·9% (92·7%-99·2%)	99·3% (98·1%-100·0%)	0·448	97·0% (95·3%-98·7%)	99·0% (98·0%-100·0%)	0·525
5-year	92·0% (86·8%-97·5%)	95·7% (92·1%-99·5%)		93·3% (90·2%-96·4%)	94·0% (91·0%-97·0%)	
10-year	86·6% (75·9%-98·8%)	88·1% (78·8%-98·5%)		84·8% (77·9%-92·2%)	84·3% (77·0%-92·3%)	
**Relapse-free survival**						
2-year	92·5% (88·3%-96·8%)	96·0% (92·8%-99·2%)	0·522	95·3% (93·2%-97·4%)	96·8% (95·1%-98·5%)	0·951
5-year	90·1% (84·9%-95·6%)	87·7% (81·5%-94·3%)		88·7% (84·9%-92·7%)	89·2% (85·6%-93·0%)	
10-year	66·6% (50·1%-88·5%)	82·0% (72·5%-92·8%)		78·4% (70·1%-87·7%)	79·0% (71·8%-86·8%)	

Among patients with stage II cancer in the ET treatment group, the median time from surgery to initiation of adjuvant ET was 28 days for those with polypharmacy and 30 days for those with an age-adjusted CCI score of 3+. For women with stage II cancer with polypharmacy or with an age-adjusted CCI score of 3+, all-cause mortality was much worse than their counterparts with stage I cancer (Fig. 4, Table 2). The 10-year all-cause mortality rate (49.0%-56.7%) were around four times the breast cancer-specific mortality rate (11.9%-15.2%) and twice the relapse rate (18.0%-33.4%) for these patients. No significant difference was identified between patients receiving ET and patients without in breast cancer-specific survival nor relapse-free survival. However, patients with an age-adjusted CCI score of 3+ and receiving ET had better all-cause survival than patients without ET, with a respective 10-year all-cause survival of 51.0% and 46.8% (p-value = 0.022). The number of patients having 10 years follow-up was small: only 28 patients with polypharmacy and 59 patients with an age-adjusted CCI score of 3+. The small number of patients with 10 years follow-up was related to the high all-cause mortality.

**Fig. 4:**
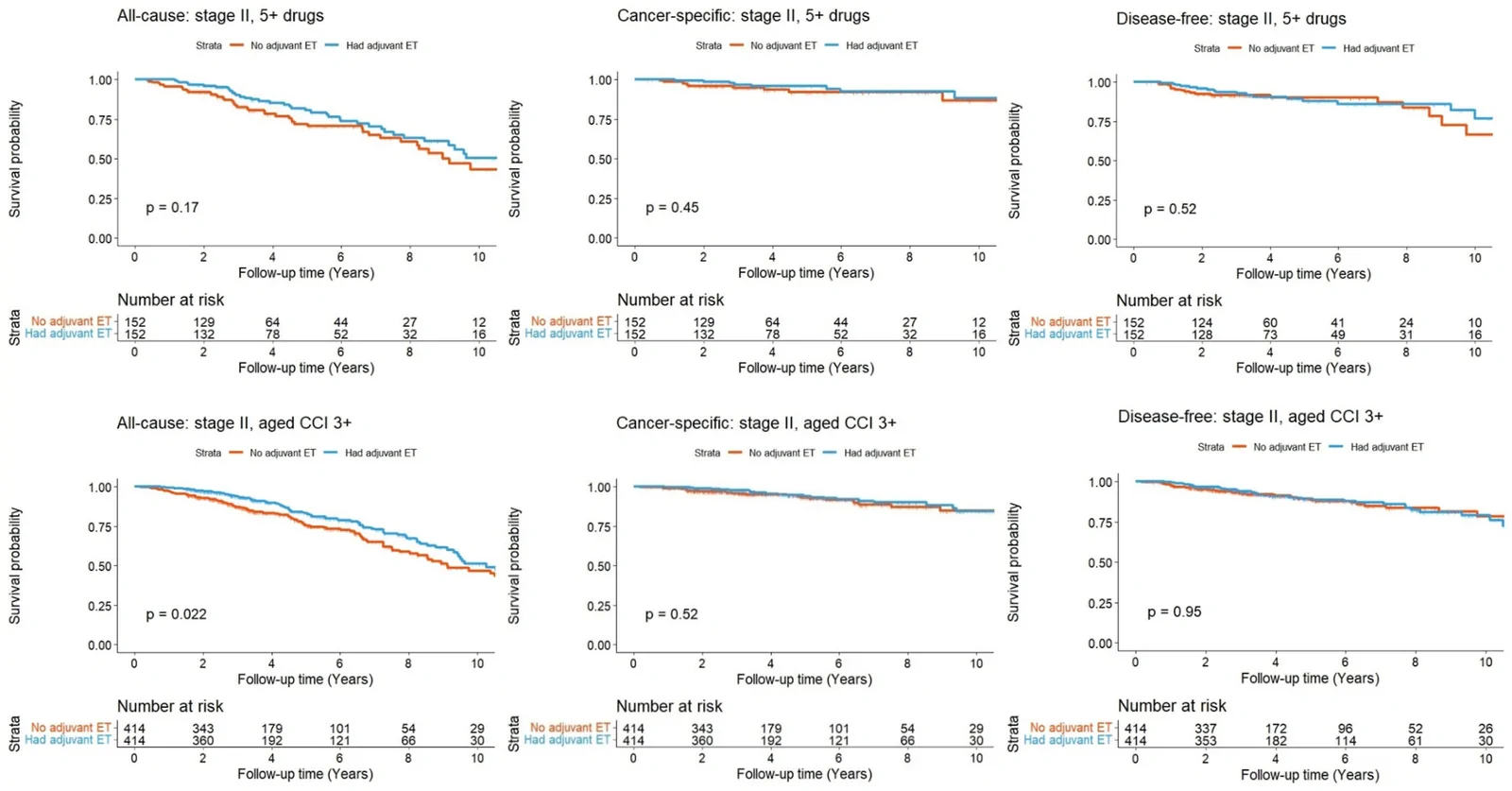
Survival in women with stage II cancer experiencing polypharmacy and women with an age adjusted CCI score 3+, comparing treatment and control groups.

## 4 Discussion

For older patients, patients with multiple comorbidities and those on numerous long-term medications, the decision to initiate adjuvant ET can be challenging. These patients often have competing health priorities and may be more susceptible to side effects. In such cases, the risk-benefit ratio might be unfavourable, and the risk of overtreatment might be significant. In addition, side effects from ET can have significant impact on breast cancer survivor's quality of life [14]. The European Society of Breast Cancer Specialists (EUSOMA) and the International Society of Geriatric Oncology (SIOG) recommended that treatment decisions in older adults should consider not only the risk of breast cancer recurrence, but also the risk of dying of other causes, which is strongly affected by frailty [15]. This study showed women with stage I and II cancer who had polypharmacy or an age-adjusted CCI score of 3+ had poorer all-cause survival. Decisions regarding the initiation of adjuvant ET in these patients should take this into consideration.

Breast cancer-specific survival and disease-free survival were excellent, but the all-cause survival was poor for stage I HR + breast cancer among women with polypharmacy or with an age-adjusted CCI score of 3+ (including all women aged 70+ years, and younger women with severe comorbidity). Among these patients, we did not find any statistically significant difference in all-cause survival, breast-cancer specific survival, disease-free survival between the adjuvant ET group and no adjuvant ET group. This finding may reflect the combination of a low risk of cancer recurrence and a high risk of competing mortality, potentially limiting the absolute survival benefit of ET. The St Gallen consensus, ASCO and European geriatric oncology all recommended that the choice and duration of adjuvant ET should be individualised according to recurrence risk, menopausal status, treatment tolerance, life expectancy, comorbidities, and patient preferences. [16], [17], [18] Treatment de-escalation is increasingly being considered for older women with low-risk HR-positive breast cancer. There are evidence supporting that radiotherapy can be omitted in older patients with lower-risk disease and personalising therapy may be more suitable for older women with early-stage HR + breast cancer [19], [20], [21]. Competing-risk estimation, genomic risk assessment and comprehensive geriatric assessment may help refine individualised decisions by integrating tumour biology, life expectancy, comorbidities, functional status and patient preferences. [19], [20], [21].

For patients with stage II disease, the treatment group had better 10-year all-cause survival than the control group though not significant. This study could not explore the outcomes amongst patients with stage III cancer because of the small number of patients not having adjuvant ET in this group. However, previous evidence showed that women with node-positive breast cancer derive greater absolute benefit from adjuvant endocrine therapy compared to patients with node-negative cancer [22], [23]. Therefore, we believe women with stage III hormone receptor positive breast cancer with polypharmacy or with an age-adjusted CCI score 3+ benefit from adjuvant ET.

Only 54.7% of women completed five years of adjuvant ET in our study, which was lower than the pooled mean persistence rate of 66.8% reported in a systematic review of 26 studies [24]. The discrepancy may be able to be explained by the differences in populations and outcome definitions [24]. Perceived adjuvant ET importance and trust in doctor were found to be associated with greater persistence, and doubts about efficacy were associated with lower persistence [24], [25]. The lower persistence rate of the five years of adjuvant ET among women with stage III cancer was associated with the higher relapse rate and comorbidities in this group of patients, with 22.2% discontinued ET because of relapse and 5.1% because of deaths from other causes.

Using population-based real-world data, this study provides detailed information on the duration/adherence of adjuvant ET, and outcomes of elderly women on ET with comorbidity and polypharmacy. These patients comprise a big proportion of the breast cancer patient population, but they are not often included in clinical trials. The NZBCR records comprehensive data on patient treatment and tumour characteristics and the linkage to the national administrative datasets improved completeness of the data.

This study has some limitations. The observational design of this study limits causal inference due to potential residual confounding and selection bias. The decision to initiate or withhold ET in routine practice is highly selective. Although we have excluded patients who were deeded not fit for ET, there are other factors such as functional status, anticipated life expectancy and clinician judgment were not recorded in the dataset. For survival analysis among patients with stage II cancer, the number of patients with 10+ years follow-up was small and therefore the results were not robust. In the propensity score matching, we have stratified patients by cancer stage and have included the important variables (e.g. tumour characteristics and other treatments), but there might be still other confounding factors not included (e.g. body mass index and frailty). The duration and adherence to adjuvant ET were estimated using dispensing data from the PHARMS dataset, but we could not confirm whether patients had taken the medications as prescribed.

## 5 Conclusions

Breast cancer-specific survival and disease-free survival were excellent for stage I hormone receptor positive breast cancer among women with old age, polypharmacy, or comorbidities. No difference was identified between patients receiving adjuvant ET and patients without. These patients might not be able to benefit from adjuvant ET. Further research should be performed to verify these findings.

## CRediT authorship contribution statement

**Chunhuan Lao:** Writing – review & editing, Writing – original draft, Software, Resources, Project administration, Methodology, Investigation, Funding acquisition, Formal analysis, Data curation, Conceptualization. **Marion Kuper-Hommel:** Writing – review & editing, Validation, Supervision, Methodology, Investigation, Conceptualization. **Ian Campbell:** Writing – review & editing, Validation, Supervision, Methodology, Investigation, Conceptualization. **Ineke Meredith:** Writing – review & editing, Investigation, Formal analysis, Conceptualization. **Abbey Wrigley:** Writing – review & editing, Methodology, Formal analysis, Conceptualization. **Ross Lawrenson:** Writing – review & editing, Validation, Supervision, Methodology, Funding acquisition, Formal analysis, Conceptualization.

## Data sharing

No data from this study will be shared. Data used in this study will be available from the Te Rēhita Mate Ūtaetae Breast Cancer Foundation National Register and Health New Zealand with ethics approval.

## Declaration of competing interest

The authors declare the following financial interests/personal relationships which may be considered as potential competing interests: Chunhuan Lao reports financial support was provided by Health Research Council of New Zealand. If there are other authors, they declare that they have no known competing financial interests or personal relationships that could have appeared to influence the work reported in this paper.

## Figures and Tables

**Table:** Appendix Table 1Specific chemicals excluded in the polypharmacy estimation

Therapeutic group	Details
Therapeutic group 1	Dermatologicals
Therapeutic group 1	Special foods
Therapeutic group 1	Sensory organs
Therapeutic group 1	Extemporaneously compounded preparations and galenicals
Therapeutic group 1	National immunisation schedule
Therapeutic group 2	Respiratory devices
Therapeutic group 2	Unknown (eg, header rows, named patient authority)
Therapeutic group 2	Various (eg, brand switch fee)
Therapeutic group 2	Diabetes management: ketone testing, blood glucose testing, insulin syringes and needles, insulin pumps, insulin pump consumables

**Appendix Table 2:** Patient characteristics and treatments in women with stage I cancer and taking 5+ long-term drugs

Factors		Before matching				After matching			
		No adjuvant endocrine therapy	Had adjuvant endocrine therapy	p-value	SMD	No adjuvant endocrine therapy	Had adjuvant endocrine therapy	p-value	SMD
**Number of patients**		460	577			299	299		
**Number of long-term drugs (mean (SD))**		6.83 (2.22)	6.54 (2.01)	0.030	0.135	6.65 (1.90)	6.58 (2.14)	0.657	0.022
**Unadjusted CCI score (mean (SD))**		0.59 (1.17)	1.25 (1.58)	<0.001	0.473	0.75 (1.38)	0.79 (1.17)	0.702	0.024
**Year of diagnosis (mean (SD))**		2017.10 (4.13)	2016.60 (4.14)	0.054	0.120	2016.80 (4.27)	2016.90 (4.00)	0.767	0.060
**Age at diagnosis (mean (SD))**		68.24 (9.70)	67.55 (9.45)	0.253	0.071	67.91 (9.85)	68.25 (9.14)	0.661	0.027
**Menopausal status (%)**	Pre-menopausal	13 (2.8)	22 (3.8)	0.525	0.071	9 (3.0)	9 (3.0)	0.844	0.145
	Peri-menopausal	11 (2.4)	10 (1.7)			7 (2.3)	5 (1.7)		
	Post-menopausal	436 (94.8)	545 (94.5)			283 (94.6)	285 (95.3)		
**Ethnicity (%)**	European/Others	349 (75.9)	422 (73.1)	0.016	0.203	228 (76.3)	234 (78.3)	0.892	0.056
	Māori	44 (9.6)	86 (14.9)			35 (11.7)	34 (11.4)		
	Pacific	29 (6.3)	40 (6.9)			18 (6.0)	14 (4.7)		
	Asian	38 (8.3)	29 (5.0)			18 (6.0)	17 (5.7)		
**HER2 status (%)**	Negative	441 (95.9)	520 (90.1)	0.001	0.232	284 (95.0)	283 (94.6)	1.000	0.015
	Positive	19 (4.1)	57 (9.9)			15 (5.0)	16 (5.4)		
**Cancer grade (%)**	1	222 (48.3)	157 (27.2)	<0.001	0.472	119 (39.8)	115 (38.5)	0.859	0.036
	2	199 (43.3)	318 (55.1)			148 (49.5)	148 (49.5)		
	3	39 (8.5)	102 (17.7)			32 (10.7)	36 (12.0)		
**Tumour Size (mm, mean (SD))**		10.38 (4.58)	13.07 (4.40)	<0.001	0.599	11.56 (4.56)	11.31 (4.48)	0.508	0.012
**Type Of breast surgery (%)**	Breast conserving surgery	347 (75.4)	444 (76.9)	0.620	0.036	233 (77.9)	230 (76.9)	0.845	0.033
	Mastectomy	113 (24.6)	133 (23.1)			66 (22.1)	69 (23.1)		
**Radiotherapy (%)**	No	186 (40.4)	211 (36.6)	0.227	0.080	115 (38.5)	124 (41.5)	0.504	0.028
	Yes	274 (59.6)	366 (63.4)			184 (61.5)	175 (58.5)		
**Chemotherapy (%)**	No	413 (89.8)	502 (87.0)	0.199	0.087	267 (89.3)	268 (89.6)	1.000	0.021
	Yes	47 (10.2)	75 (13.0)			32 (10.7)	31 (10.4)		
**Biological therapy (%)**	No	454 (98.7)	550 (95.3)	0.004	0.199	293 (98.0)	291 (97.3)	0.787	0.053
	Yes	6 (1.3)	27 (4.7)			6 (2.0)	8 (2.7)		

**Appendix Table 3:** Patient characteristics and treatments in women with stage I cancer and age-adjusted CCI 3+

Factors		Before matching				After matching			
		No adjuvant endocrine therapy	Had adjuvant endocrine therapy	p-value	SMD	No adjuvant endocrine therapy	Had adjuvant endocrine therapy	p-value	SMD
**Number of patients**		1027	1346			743	743		
**Number of long-term drugs (mean (SD))**		3.05 (2.80)	3.21 (2.82)	0.155	0.059	3.12 (2.85)	2.99 (2.66)	0.381	0.008
**Unadjusted CCI score (mean (SD))**		0.74 (1.33)	1.35 (1.65)	<0.001	0.412	0.86 (1.47)	0.90 (1.23)	0.632	0.009
**Year of diagnosis (mean (SD))**		2017.78 (3.85)	2017.26 (3.89)	0.001	0.135	2017.66 (3.91)	2017.64 (3.72)	0.897	0.009
**Age at diagnosis (mean (SD))**		73.65 (7.36)	70.80 (8.45)	<0.001	0.360	73.08 (7.70)	73.08 (7.48)	0.984	0.008
**Menopausal status (%)**	Pre-menopausal	3 (0.3)	26 (1.9)	<0.001	0.183	2 (0.3)	4 (0.5)	0.605	0.066
	Peri-menopausal	1 (0.1)	9 (0.7)			1 (0.1)	2 (0.3)		
	Post-menopausal	1023 (99.6)	1311 (97.4)			740 (99.6)	737 (99.2)		
**Ethnicity (%)**	European/Others	887 (86.4)	1038 (77.1)	<0.001	0.279	620 (83.4)	634 (85.3)	0.646	0.061
	Māori	61 (5.9)	177 (13.2)			57 (7.7)	52 (7.0)		
	Pacific	27 (2.6)	63 (4.7)			21 (2.8)	22 (3.0)		
	Asian	52 (5.1)	68 (5.1)			45 (6.1)	35 (4.7)		
**HER2 status (%)**	Negative	980 (95.4)	1202 (89.3)	<0.001	0.230	700 (94.2)	695 (93.5)	0.665	0.023
	Positive	47 (4.6)	144 (10.7)			43 (5.8)	48 (6.5)		
**Cancer grade (%)**	1	465 (45.3)	283 (21.0)	<0.001	0.547	242 (32.6)	238 (32.0)	0.874	0.042
	2	467 (45.5)	822 (61.1)			408 (54.9)	417 (56.1)		
	3	95 (9.3)	241 (17.9)			93 (12.5)	88 (11.8)		
**Tumour Size (mm, mean (SD))**		11.49 (4.80)	13.40 (4.43)	<0.001	0.413	12.38 (4.73)	12.34 (4.61)	0.886	0.017
**Type Of breast surgery (%)**	Breast conserving surgery	732 (71.3)	969 (72.0)	0.736	0.016	530 (71.3)	521 (70.1)	0.648	0.027
	Mastectomy	295 (28.7)	377 (28.0)			213 (28.7)	222 (29.9)		
**Radiotherapy (%)**	No	497 (48.4)	560 (41.6)	0.001	0.137	340 (45.8)	350 (47.1)	0.640	0.027
	Yes	530 (51.6)	786 (58.4)			403 (54.2)	393 (52.9)		
**Chemotherapy (%)**	No	943 (91.8)	1199 (89.1)	0.031	0.093	682 (91.8)	677 (91.1)	0.711	0.005
	Yes	84 (8.2)	147 (10.9)			61 (8.2)	66 (8.9)		
**Biological therapy (%)**	No	1012 (98.5)	1276 (94.8)	<0.001	0.210	729 (98.1)	726 (97.7)	0.717	0.010
	Yes	15 (1.5)	70 (5.2)			14 (1.9)	17 (2.3)		

**Appendix Table 4:** Patient characteristics and treatments in women with stage II cancer and taking 5+ long-term drugs

Factors		Before matching				After matching			
		No adjuvant endocrine therapy	Had adjuvant endocrine therapy	p-value	SMD	No adjuvant endocrine therapy	Had adjuvant endocrine therapy	p-value	SMD
**Number of patients**		179	686			152	152		
**Number of long-term drugs (mean (SD))**		6.89 (1.97)	6.70 (1.89)	0.218	0.102	6.86 (1.91)	6.86 (2.09)	0.977	0.025
**Unadjusted CCI score (mean (SD))**		0.92 (1.44)	1.37 (1.58)	0.001	0.296	1.03 (1.52)	1.09 (1.42)	0.756	0.132
**Year of diagnosis (mean (SD))**		2017.42 (3.77)	2017.29 (3.92)	0.674	0.036	2017.45 (3.80)	2017.27 (3.84)	0.686	0.079
**Age at diagnosis (mean (SD))**		73.74 (11.97)	68.96 (11.41)	<0.001	0.409	72.68 (11.85)	72.86 (11.22)	0.893	0.059
**Menopausal status (%)**	Pre-menopausal	12 (6.7)	48 (7.0)	0.706	0.075	10 (6.6)	7 (4.6)	0.632	0.052
	Peri-menopausal	2 (1.1)	14 (2.0)			2 (1.3)	1 (0.7)		
	Post-menopausal	165 (92.2)	624 (91.0)			140 (92.1)	144 (94.7)		
**Ethnicity (%)**	European/Others	145 (81.0)	493 (71.9)	0.088	0.226	120 (78.9)	118 (77.6)	0.987	0.112
	Māori	16 (8.9)	103 (15.0)			16 (10.5)	16 (10.5)		
	Pacific	10 (5.6)	47 (6.9)			8 (5.3)	9 (5.9)		
	Asian	8 (4.5)	43 (6.3)			8 (5.3)	9 (5.9)		
**HER2 status (%)**	Negative	165 (92.2)	597 (87.0)	0.077	0.158	138 (90.8)	139 (91.4)	1.000	0.023
	Positive	14 (7.8)	89 (13.0)			14 (9.2)	13 (8.6)		
**Cancer grade (%)**	1	31 (17.3)	101 (14.7)	0.603	0.083	26 (17.1)	31 (20.4)	0.712	0.088
	2	94 (52.5)	385 (56.1)			81 (53.3)	75 (49.3)		
	3	54 (30.2)	200 (29.2)			45 (29.6)	46 (30.3)		
**Tumour Size (mm, mean (SD))**		29.98 (11.47)	28.53 (12.41)	0.158	0.121	29.07 (10.22)	30.15 (15.20)	0.470	0.067
**Type Of breast surgery (%)**	Breast conserving surgery	69 (38.5)	312 (45.5)	0.114	0.141	60 (39.5)	63 (41.4)	0.815	0.092
	Mastectomy	110 (61.5)	374 (54.5)			92 (60.5)	89 (58.6)		
**Radiotherapy (%)**	No	113 (63.1)	309 (45.0)	<0.001	0.369	88 (57.9)	92 (60.5)	0.726	0.039
	Yes	66 (36.9)	377 (55.0)			64 (42.1)	60 (39.5)		
**Chemotherapy (%)**	No	151 (84.4)	523 (76.2)	0.026	0.205	126 (82.9)	130 (85.5)	0.637	0.017
	Yes	28 (15.6)	163 (23.8)			26 (17.1)	22 (14.5)		
**Biological therapy (%)**	No	176 (98.3)	642 (93.6)	0.021	0.242	149 (98.0)	148 (97.4)	1.000	0.114
	Yes	3 (1.7)	44 (6.4)			3 (2.0)	4 (2.6)		

**Appendix Table 5:** Patient characteristics and treatments in women with stage II cancer and age-adjusted CCI 3+

Factors		Before matching				After matching			
		No adjuvant endocrine therapy	Had adjuvant endocrine therapy	p-value	SMD	No adjuvant endocrine therapy	Had adjuvant endocrine therapy	p-value	SMD
**Number of patients**		434	1785			414	414		
**Number of long-term drugs (mean (SD))**		3.32 (2.92)	3.20 (2.79)	0.392	0.045	3.28 (2.93)	3.23 (2.72)	0.806	0.020
**Unadjusted CCI score (mean (SD))**		0.95 (1.67)	1.20 (1.58)	0.003	0.155	0.97 (1.70)	1.05 (1.51)	0.489	0.035
**Year of diagnosis (mean (SD))**		2018.02 (3.61)	2017.02 (4.09)	<0.001	0.259	2017.96 (3.66)	2017.89 (3.62)	0.789	0.052
**Age at diagnosis (mean (SD))**		77.05 (8.01)	72.76 (8.55)	<0.001	0.518	76.47 (7.65)	76.11 (7.74)	0.507	0.015
**Menopausal status (%)**	Pre-menopausal	4 (0.9)	26 (1.5)	0.122	0.135	4 (1.0)	7 (1.7)	0.544	0.026
	Peri-menopausal	0 (0.0)	14 (0.8)			0 (0.0)	0 (0.0)		
	Post-menopausal	430 (99.1)	1745 (97.8)			410 (99.0)	407 (98.3)		
**Ethnicity (%)**	European/Others	363 (83.6)	1373 (76.9)	0.012	0.189	346 (83.6)	334 (80.7)	0.690	0.042
	Māori	33 (7.6)	230 (12.9)			31 (7.5)	36 (8.7)		
	Pacific	19 (4.4)	87 (4.9)			18 (4.3)	24 (5.8)		
	Asian	19 (4.4)	95 (5.3)			19 (4.6)	20 (4.8)		
**HER2 status (%)**	Negative	388 (89.4)	1597 (89.5)	1.000	0.004	370 (89.4)	362 (87.4)	0.447	0.060
	Positive	46 (10.6)	188 (10.5)			44 (10.6)	52 (12.6)		
**Cancer grade (%)**	1	67 (15.4)	252 (14.1)	0.641	0.050	62 (15.0)	53 (12.8)	0.664	0.080
	2	240 (55.3)	1029 (57.6)			233 (56.3)	239 (57.7)		
	3	127 (29.3)	504 (28.2)			119 (28.7)	122 (29.5)		
**Tumour Size (mm, mean (SD))**		30.06 (11.81)	29.08 (12.03)	0.124	0.083	30.15 (11.93)	30.39 (11.47)	0.769	0.036
**Type Of breast surgery (%)**	Breast conserving surgery	165 (38.0)	770 (43.1)	0.060	0.104	156 (37.7)	141 (34.1)	0.310	0.081
	Mastectomy	269 (62.0)	1015 (56.9)			258 (62.3)	273 (65.9)		
**Radiotherapy (%)**	No	273 (62.9)	815 (45.7)	<0.001	0.351	253 (61.1)	265 (64.0)	0.430	0.055
	Yes	161 (37.1)	970 (54.3)			161 (38.9)	149 (36.0)		
**Chemotherapy (%)**	No	370 (85.3)	1461 (81.8)	0.109	0.092	353 (85.3)	344 (83.1)	0.446	0.020
	Yes	64 (14.7)	324 (18.2)			61 (14.7)	70 (16.9)		
**Biological therapy (%)**	No	420 (96.8)	1694 (94.9)	0.128	0.094	400 (96.6)	395 (95.4)	0.477	0.038
	Yes	14 (3.2)	91 (5.1)			14 (3.4)	19 (4.6)		
